# Statistical literacy and scientific reasoning & argumentation in physicians

**DOI:** 10.3205/zma001473

**Published:** 2021-04-15

**Authors:** Felicitas M. Schmidt, Jan M. Zottmann, Maximilian Sailer, Martin R. Fischer, Markus Berndt

**Affiliations:** 1 University Hospital, LMU Munich, Institute of Medical Education, Munich, Germany; 2 University of Passau, Chair of Educational Science, Passau, Germany; 3 Walden University, Richard W. Riley College of Education and Leadership, Minneapolis, USA

**Keywords:** evidence-based practice, scientific reasoning and argumentation, statistical literacy, medical education, postgraduate medical education

## Abstract

**Objective: **Statistical literacy (SL) of physicians, i.e. the ability to use and interpret statistical numbers in the context of science, is an essential prerequisite for risk estimation and communication. Together with scientific reasoning and argumentation (SRA) skills, SL provides the basis for evidence-based practice. Several studies suggest that in medical students both skills are underdeveloped. The aim of the present study was to investigate these skills in practicing physicians and how these skills were acquired.

**Methods: **Data collection in *N*=71 physicians was conducted online and as paper pencil. SL was assessed with multiple-choice items. SRA skills evidence evaluation and drawing conclusions were measured with a decision scenario.

**Results: **Study results indicated that physicians have medium levels of SL (*M*=17.58, *SD*=6.92, max 30 pts.) and SRA (evidence evaluation: *M*=7.75, *SD*=1.85, max 10 pts.; drawing conclusions: *M*=37.20, *SD*=5.35, max 60 pts.). Skills development via autodidactic learning activities (*M*=4.78, *SD*=1.13, range 1-6) was reported significantly more often than development during formal medical education (*M*=2.31, *SD*=1.46), *t*(71)=-9.915, *p*<.001, or in extracurricular activities (*M*=3.34, *SD*=1.87), *t*(71)=4.673, *p*<.001. The active involvement in research seemed decisive: The number of publications and time spent in research significantly correlated with SL, *r*(71)=.355, *p*=.002; respectively *r*(71)=.280, *p*=.018. SRA skills were predicted by the type of MD-thesis, *β*=-.380, *p*=.016, and working in research, *β*=3.355, *p*=.008.

**Conclusion: **Active involvement in research activities seems to be a very important factor for the development of both SL and SRA skills. The implementation of systematic fostering of these skills during formal medical education seems warranted.

## Zusammenfassung

**Ziel: **Statistikverständnis (SV) von ÄrztInnen, d. h. die Fähigkeit statistische Zahlen im wissenschaftlichen Kontext zu verwenden und zu interpretieren, ist eine wichtige Voraussetzung für Risikoabschätzung und -kommunikation. Statistikverständnis bildet in Verbindung mit der Fähigkeit zum wissenschaftlichen Denken und Argumentieren (WDA) die Grundlage für eine evidenzbasierte medizinische Praxis. Mehrere Studien legen nahe, dass beide Fähigkeiten bei Medizinstudierenden nicht ausreichend entwickelt sind. Ziel der vorliegenden Studie war die Untersuchung dieser Fähigkeiten bei praktizierenden ÄrztInnen und wie diese Fähigkeiten erworben wurden.

**Methoden: **Die Daten von *N*=71 ÄrztInnen wurden online und in Papierform erhoben. Statistikverständnis wurde anhand von Multiple-Choice-Fragen gemessen, die WDA-Aktivitäten Evidenzen bewerten und Schlussfolgerungen ziehen anhand eines Entscheidungsszenarios.

**Ergebnisse: **Die Studienergebnisse zeigten bei ÄrztInnen ein mittleres Niveau im SV (*M*=17.58, *SD*=6.92, max. 30 Pkt.) und WDA (Evidenzen bewerten: *M*=7.75, *SD*=1.85, max. 10 Pkt.; Schlussfolgerungen ziehen: *M*=37.20, SD=5.35, max. 60 Pkt.). Die Entwicklung entsprechender Fähigkeiten durch autodidaktische Lernaktivitäten (*M*=4.78, *SD*=1.13, Spannweite 1–6) wurde signifikant häufiger angegeben als während der formalen medizinischen Ausbildung (*M*=2.31, *SD*=1.46), *t*(71)=-9.915, *p*<.001 oder durch außercurriculäre Aktivitäten (*M*=3.34, *SD=1.87), t*(71)=4.673, *p*<.001. Die aktive Beteiligung an Forschung schien eine entscheidende Rolle zu spielen: Die Anzahl der Veröffentlichungen und die Zeit der Tätigkeit in der Forschung korrelierten signifikant mit dem SV, *r*(71)=.355, *p*=.002; respektive *r*(71)=.280, *p*=.018. WDA-Fähigkeiten wurden vorhergesagt durch die Art der medizinischen Doktorarbeit, *β*=-.380, *p*=.016, sowie durch die Tätigkeit in der Forschung, *β*=3.355, *p*=.008.

**Schlussfolgerung: **Das aktive Mitwirken an Forschungsaktivitäten scheint ein sehr wichtiger Faktor für die Entwicklung sowohl des SV als auch des WDA zu sein. Dies spricht für die Implementierung einer systematischen Förderung dieser Fähigkeiten in die formale medizinische Ausbildung.

## 1. Background

Following Sackett et al. (1997) regarding the modern definition of evidence-based medicine, scientific reasoning skills are considered essential together with physicians’ expertise for best possible decision-making in the best interest of the patient [1], [2], [3]. For the assessment of risks and probabilities and their applicability to specific patients, a basic understanding of statistics and evidence evaluation is necessary. Statistical literacy (SL) is not only the ability to understand statistical information, but also to apply it in decision-making [4]. It comprises the aptitude of critical reflection about statistics as evidence in arguments [5]. Statistical literacy is based on numeracy, the aptitude of mathematical operations [6], and encompasses the ability to use and interpret statistical numbers in the context of science [7], [8], [9] and the ability to explain and critically evaluate them [10], [11] (working definition for the present study). Furthermore, it is intertwined with scientific reasoning and argumentation skills (SRA), to provide the basis for evidence-based decision-making [12], [13]. 

Based on the framework by Fischer et al. (2014), SRA can be defined as the competence of comprehending and applying scientific working methods and their results when solving problems [14], [15]. SRA can be described by eight epistemic activities and this study focuses on two of them, evidence evaluation (EE) and drawing conclusions (DC).

Various studies suggest an intertwining of SRA and SL, with Anderson et al. (2013) stating that the latter is needed to evaluate scientific evidence [16] and Franklin et al. (2005) hypothesizing that SL itself encompasses also SRA skills [17]. 

However, a collective statistical illiteracy has been observed among physicians [4], [18]. Similarly, SRA skills needed for evidence-based practice [19], such as EE or DC, are underdeveloped [4], [7]. 

In a study by Anderson et al. (2014), 52% of the participating physicians answered only two (or fewer) of four questions regarding statistical concepts correctly [7]. This is in line with findings by Windish et al. (2007) reporting only 40% of resident physicians demonstrating adequate understanding of biostatistical concepts [20]. Similar results were found by Gigerenzer and Wegwarth (2008), showing that 33% of gynecologists were not aware of the benefits of mammography screening, with 79% being unable to interpret the positive predictive value [21]. Gigerenzer et al. (2008) summarized various studies on the concept of the positive predictive value and its dependence on prevalence, finding that 50% of participants were under the impression that false positive test results in HIV testing do not exist. They also found that only two of 20 urologists have sufficient knowledge about the reliability of a PSA-test. Thus, a lot of measurement tools for the assessment of SL exist [18], [22], [23], which focus commonly on one of the three levels of Watson (1997) or are designed in a certain context [7], [22]. Overall, physicians’ SL is not below-average [7]. However, it can be considered comparable to other educated samples [18], [24] and was found superior to that of residents in research training [20] or medical students [25]. The few studies that have assessed medical students’ SL are supporting the findings of superiority of physicians [26]. Berndt et al. (2021) compared medical students to those of social sciences and economics and found that medical students in their first years of study scored higher in comparison to social science students and comparable to economics students [27]. This study not only assessed SL, but also the two aforementioned skills EE and DC. Further possible links between SL and SRA have been examined with the Medical Data Interpretation Test [22] where participating physicians scored higher overall than participants with other postgraduate degrees (89 out of 100 score points). Johnson et al. (2014) assessed numeracy of medical students and residents and found students with poor numeracy being more likely to misjudge risks of different treatment alternatives and that the confidence in treatment recommendation increased during medical school [25]. 

In summary, SL and SRA seem to be underdeveloped in medical students and better developed in physicians. However, the development of SL and SRA may not necessarily happen within formal education and the question remains how, where, and when this development occurs. The present study should contribute to the discussion when and how to best foster SL and SRA skills in lifelong learning of physicians by applying a test instrument comprising various aspects of SL and SRA. It aimed at providing further insights into these skills in physicians and to identify demographic factors and learning opportunities that may be associated with the development of these skills. Our explorative research questions were:

**RQ1. Statistical literacy and SRA skills**

a. To what extent are SL and SRA skills developed in physicians? 

b. To what extent does SL predict SRA skills of physicians?

**RQ2. Education and skills development**

a. How, where, and when do physicians develop SL and SRA skills? 

b. Which demographic factors are related to the development of SL and SRA skills?

## 2. Methods

### 2.1. Design and sample

Our study followed a quasi-experimental, causal-comparative design with two dependent variables: SL and SRA. We included *N*=71 German-speaking physicians (31 females, 34 males, 6 NA), from different work settings and locations in our study: hospital (*n*=44), outpatient sector (*n*=3), research (*n*=8), study program (*n*=2) and administration (*n*=2), (*n*=12 NA). A MD-thesis, a scientific work as optional part of the medical study program (not equivalent to a PhD thesis), was completed by 58 participants and 9 were currently working on it. Despite the modest sample size, we consider our sample representative with regard to scientific experience, as a MD-thesis is very common in Germany. The mean age of participants was 40 years (*SD*=9.59, range=26-65) (see table 1 (Tab. 1)).

#### 2.2. Test instrument

For the assessment of SL and SRA skills, we used an instrument previously developed in the context of a study by Berndt et al. (2021) who initiated the ongoing validation process with 217 economics, social sciences, and medical students from LMU Munich [27]. The test instrument combines multiple choice items to assess SL with a decision scenario [28] to assess the participants’ skills in EE and DC. For this study, items on relevant demographic factors were added and piloted with ten medical students from LMU Munich.

##### 2.2.1. Demography

Demographic and biographic parameters of the participants were assessed with a special interest in their working history and environment (hospital, out-patient care, research). Questions were adapted from a study by Epstein et al. [29] and comprised multiple choice items, some with the opportunity to fill in additional free text; five items on the MD-thesis, three items on the professional career, two items on the publication record (type of authorship, number of publications), and three items on the current job description. 

##### 2.2.2. Statistical literacy 

Statistical literacy was measured with multiple choice items based on validated instruments [7], [18], [23], to assess a broad spectrum from basic numeracy to conditional probabilities and statistical concepts. Duplicates and factual knowledge questions were excluded, so that all three levels described by Watson (1997) were covered. Additionally, all items were weighted for difficulty [11]. Internal consistency of the SL test was .82 (Cronbach’s α) in our sample with a maximum score of 30 points. All items were framed in a medical context; however, no medical content knowledge was necessary to answer them correctly. 

##### 2.2.3. SRA skills 

The assessment of SRA skills focused on the two epistemic activities EE and DC with a decision scenario in a medical context (general medicine, out-patient care) and provided two separate overall scores for EE and DC (Cronbach's α for EE items .87; for DC items .74). For EE, participants had to evaluate four pieces of evidence [30], [31], [32], including one authentic pharmaceutical brochure that advertised herbal drugs, in terms of their scientific quality, evidence strength, and relevance for the present situation on a 6-point Likert scale based on the QUESTS criteria [33].

Then, the participants rated the persuasiveness (Likert 1-6) of 20 arguments, which were extracted from the presented evidence and assigned a level of argument strength from 1 (lowest) to 4 (highest). For 13 participants, the evaluation of 1 to 5 arguments out of 20 was missing. In order to avoid dropouts of these cases, the respective values were imputed from the average of the respective item. The participants’ ratings for scientific quality were compared to the ratings of scientific quality by the authors, resulting in a measure of similarity for EE and DC. The range of these measures was from 0-10 (EE score) and 0-60 (DC score) with zero indicating no similarity. 

#### 2.3. Procedure

The study was completed by the participants either online with LamaPoll [https://www.lamapoll.de/], a survey tool optimized for mobile applications, or as paper pencil (return rate 16.5% online and 66.7% paper pencil). Average duration was approximately 45 minutes. Participants were invited via mailing lists and personal contacts.

#### 2.4. Statistical analyses

Statistical analyses were performed with IBM SPSS 25. Descriptive and frequency data were computed for primary analysis and Cronbach’s alpha for internal consistency. Extensive outlier analyses were conducted and all required prerequisites for statistical analyses, such as normal distribution and homoscedasticity, were tested. *T*-tests, one-factorial ANOVAs, and linear regression models were calculated to assess differences and the association of demographic factors with SL and SRA. Probability values less than .05 were considered significant. Data in natural verbal language (free text in demography section) underwent independent thematic analysis by two authors to extract common themes.

## 3. Results

We included 71 completed questionnaires (see table 1 (Tab. 1)). The entire data set was checked for univariate outliers. Skewness and kurtosis for all variables was within the ±2 range [34]. The prerequisites for *t*-tests and ANOVA were fulfilled, unless indicated otherwise below. 

### 3.1. Statistical literacy and SRA skills 

The 71 physicians' average score in SL was *M*=17.58, *SD*=6.92, with a range of 5 to 30 out of 30 attainable points (59%). On average, physicians evaluated the evidences concordantly with the authors' evaluation, EE score: *M*=7.75, *SD*=1.85 (77%). The ratings for argument quality were in accordance with the authors’ rating, DC score: *M*=37.20, *SD*=5.35 (62%). Statistical literacy and DC were significantly inversely correlated, *r*(71)=-.272, *p*=.022. However, no correlation was found between SL and EE, *r*(71)=.198, *p*=.098, nor EE and DC, *r*(71)=.138, *p*=.256.

#### 3.2. Education and skills development 

**Figure 1:**
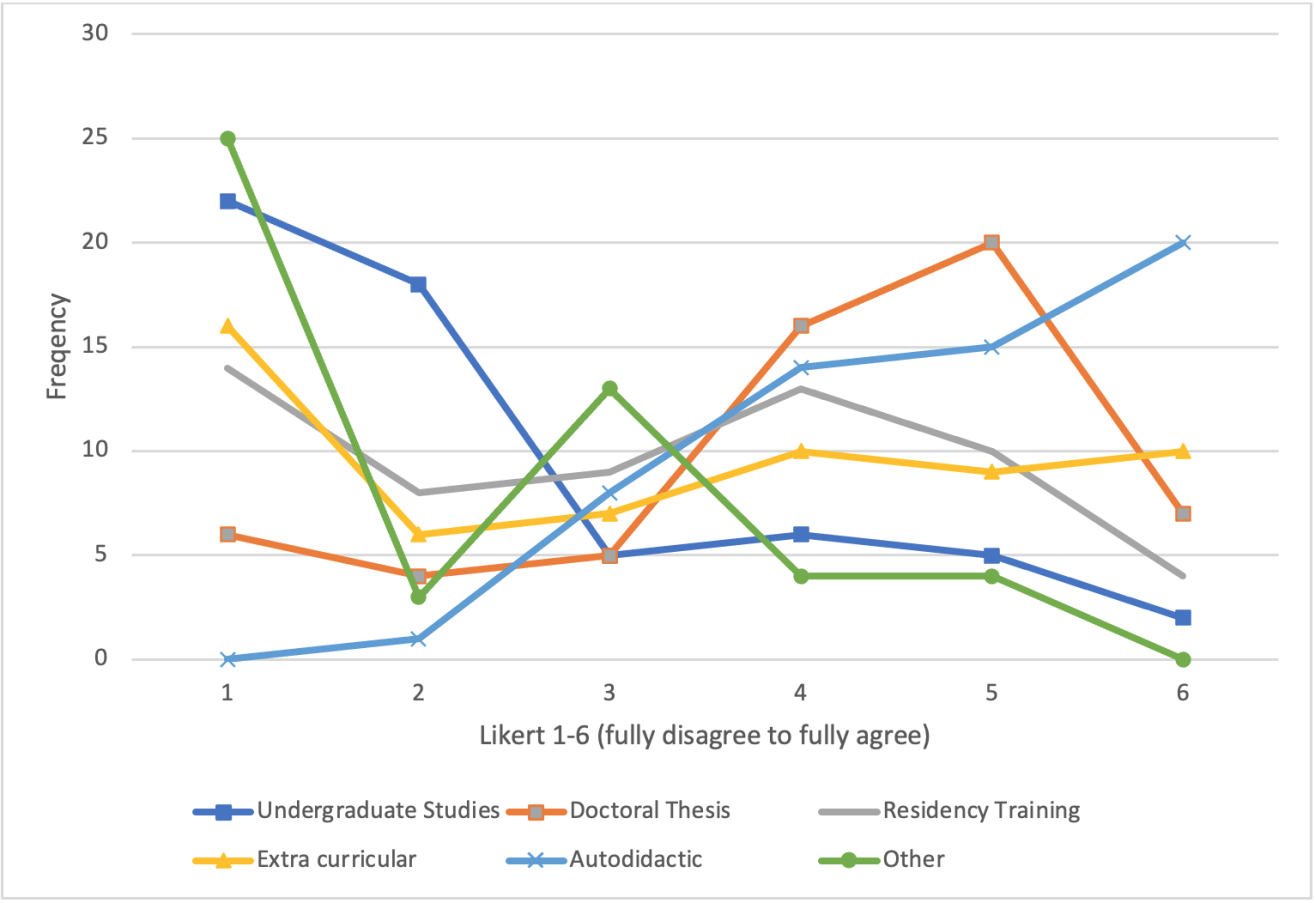
In which context have you acquired scientific skills? (*N*=71 physicians)

**Figure 2:**
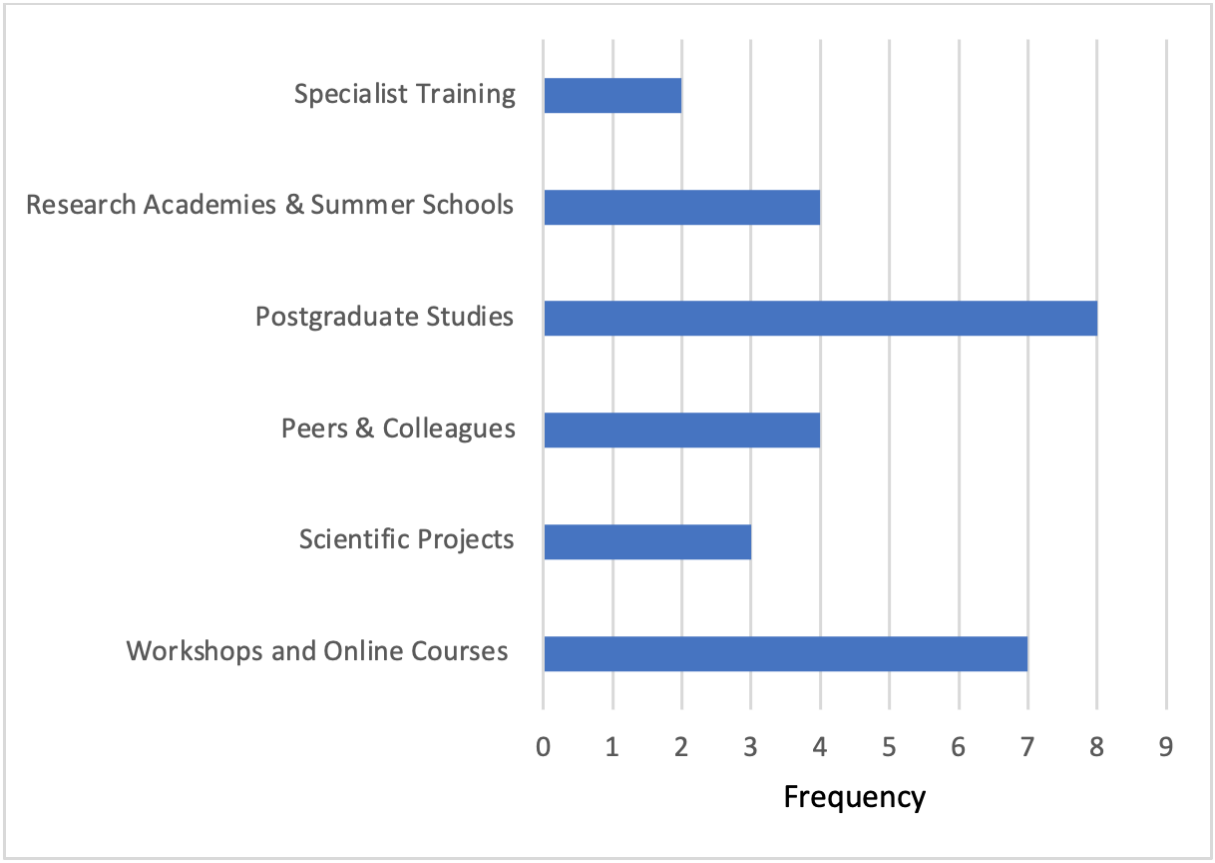
In which context have you acquired scientific skills? Free text analysis category Other (*n*=28 physicians)

We explored how, where, and when physicians developed scientific skills (see figure 1 (Fig. 1)). Significantly more participants indicated to have acquired scientific skills in an autodidactic manner (*M*=4.78, *SD*=1.13, Likert 1-6 scale) rather than during their study program (*M*=2.31, *SD*=1.46, Likert 1-6 scale),* t*(71)=-9.915, *p*<.001, or in extracurricular activities (*M*=3.34, *SD*=1.87, Likert 1-6 scale), *t*(71)=4.673, *p*<.001. In a free-text box, participants added various other learning opportunities, such as massive open online courses, higher education, workshops, and learning through peer reviews and feedback (see figure 2 (Fig. 2)). 

Having completed or working on the MD-thesis showed no effects on SL, EE, or DC. However, these results have to be treated carefully, as the prerequisites for ANOVA were not fulfilled in our sample with only four participants without a MD-thesis. The fostering of critical scrutiny of study results presented by other researchers during the preparation of the MD-thesis was positively correlated with SL, *r*(71)=.271, *p*=.033. 

Regarding the postgraduate phase, a one-factorial ANOVA showed a significant main effect of having worked in research on SL, *F*(1,70)=12.737, *p*=.001, partial η^2^=.156 and the type of authorship in publications, *F*(5,71)=3.886, *p*=.004, partial η^2^=.230. 

Time spent in research was significantly associated with better SL, *r*(71)=.28, *p*=.018, as was the number of publications, *r*(71)=.36, *p*=.002.

Regarding SRA, linear regression models revealed that the corresponding score in EE increased by β=.314±.150, *p*=.041, when the Likert value of the MD-thesis supervisor’s content-related support was increased by one point. Additionally, the form of the MD-thesis (experimental, clinical, empirical, statistical, or literature review) was associated with EE, with experimental and clinical design being positively related to EE skills, β=-.380±.154, *p*=.016, *R*^2^=.187, *F*(1,59)=4.353, *p*=.041. DC was higher when participants indicated to have already worked in research, β=3.355±1.229, *p*=.008, *R*^2^=.314 *F*(1,68)=7.448, *p*=.008. 

## 4. Discussion

### 4.1. Research question 1: Statistical literacy and SRA skills

We found average statistical literacy of physicians (59%), a rather high-level EE score (77%) and a medium-level DC score (62%). SL did not predict the SRA skills of physicians. 

Due to the focus on SL rather than the combination of basic numeracy and SL [27], our test instrument discriminated well, and we did not find any ceiling effects as observed in other educated samples [7], [35]. A comparison to other studies assessing SL of physicians is not easily done as every test covers a different range of SL. Schmidt et al. (2017) focused on knowledge of 18 different statistical tests among pathologists and observed a rather low level of SL [36]. Anderson et al. (2014) did not create an overall score but distinguished between fact, concept, and relation questions and found altering levels of SL [7]. A study with Greek residents also concentrated on knowledge questions and reported a rather low SL [26]. The EE and DC scores of German medical students we had previously examined with a similar instrument [27] were almost on the same level as the physicians’ scores in the present study. Riegelman and Hoveland (2012) found that residents struggled when critical reflection upon research was required [37], whereas the physicians in our study showed medium to high levels of SRA skills. 

EE and SL scores were not correlated. DC and SL were inversely correlated. In contextual frameworks, SL has been regarded as a prerequisite for SRA [38] and in a Dutch community-based study, more numerate participants showed enhanced performance in SRA due to increased evaluation of pros and cons in decision-making and evaluation of judgments [39]. As evidence has not been predominantly presented in numerical or statistical terms, the missing link of EE and SL was expected, but the antithetical relationship of DC and SL was not. Future research could incorporate statistical information in decision scenarios in order to further analyze this connection in practicing physicians. 

#### 4.2. Research question 2: Education and skills development

We explored how, where, and when physicians developed SL and SRA skills. They indicated to have acquired scientific skills mostly in an autodidactic manner, in higher education outside of their medical study program, or in extracurricular activities. 

Better SL was associated with the fostering of critical scrutiny of study results during the time spent working on the MD-thesis, in research or having worked in research, the number of publications, and the type of authorship. Our findings are in line with Schmidt et al. (2017), who found that having an advanced degree other than MD or statistic courses were positively associated with SL. A study with physicians, residents, and final year medical students in Thailand showed – not surprisingly – that having recently completed a statistical workshop led to higher SL scores [40]. However, additional courses are often hard to integrate in medical training. A study showed that 37% of American Ob-Gyn residents do not receive formal training [16], while another study with neurology residents observed a lack of acceptance for interventions on SL [41]. 

In the present study, better EE was associated with having been responsible for a research project (e.g. the MD-thesis) with experimental or clinical design and having content-related support by the supervisor. These findings are in line with the subjective impression of German medical graduates with a MD-thesis who rated their scientific skills higher compared to those working on it [29]. However, the participants in the study by Epstein et al. (2018) did not feel confident enough to conduct research on their own. This is particularly important because having already worked in research was associated with a higher SL and DC score in the present study and in Schmidt et al. (2017). Moreover, Epstein et al. (2018) found that medical graduates self-estimate their scientific skills after medical school as rather low. In the United States, only 68.1% of medical students in their final year participated in research during medical school and only 42% had (co-)authored a paper submitted for publication. It seems important that medical students become involved in research projects and the subsequent publication of findings during the completion of their MD-thesis, as this might enhance their SL and SRA skills in the long run. 

#### 4.3. Strengths and limitations 

This study built upon an innovative approach by the authors to assess SL and SRA skills in university students [27]. The inclusive approach of SL assessment allowed a better description of the actual skills. However, it comes with the disadvantage of limited comparability with prior research. As the participant group of practicing physicians is not easily recruited, we considered the sample size of *N*=71 to be satisfactory. While generalizability is potentially limited, our sample seems representative for the German-speaking medical education system which produces large numbers of medical doctoral degrees. The addition of numerous demographic variables yielded insights on how, where, and when scientific skills were acquired and helped to identify potential associated factors. 

Due to the broad age range in our sample, participants may have been exposed to different learning experiences in formal medical training and, depending on the place of study, may also have studied in reformed curricula. This could potentially have influenced their skills development and lead to further individual differences. In our study, we did not gather data on specific study programs, courses, and their descriptions, in which physicians might have acquired their skills. Already, the test instrument used in this study may be considered extensive and time consuming for physicians, as was indicated by 11 participants in their feedback. 

## 5. Conclusion

We assessed SL and SRA skills in German-speaking physicians together with a thorough analysis of demographic variables. The active involvement in research apparently plays an important role in the development of these skills and might in consequence enhance evidence-based practice. As most participants indicated to have acquired these skills post-graduate and in an autodidactic manner, we argue to formalize and intensify the acquisition of these skills in medical study programs. Medical education curricula should include more statistical training and aim to get students involved in research more often, e.g. by offering inquiry-based learning [42] where students conduct research projects independently and are fully responsible for all phases of the research process.

## Funding

This work was supported by the German Federal Ministry of Education and Research (BMBF, grant no. 01PB14004C) and an intramural grant of the Förderverein WiFoMed of the Medical Faculty of LMU Munich. 

## Acknowledgements

The authors would also like to thank Wolfgang Gaissmaier for his valuable comments and suggestions to the present study.

## Competing interests

The authors declare that they have no competing interests. 

## Figures and Tables

**Table 1:**
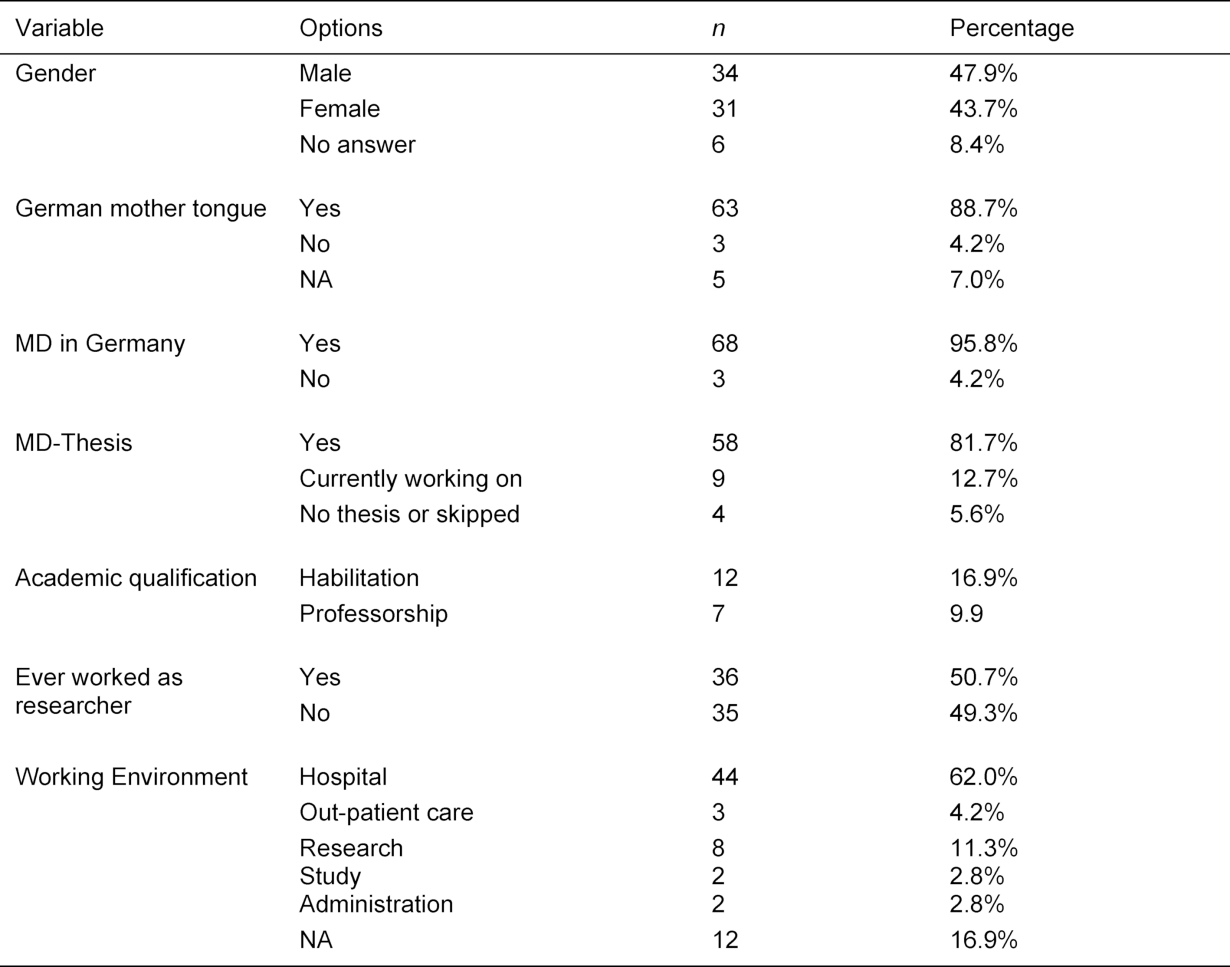
Description of study group (*N*=71)
